# Hemodynamic predictors of rupture in abdominal aortic aneurysms: a case series using computational fluid dynamics

**DOI:** 10.3389/fcvm.2025.1633938

**Published:** 2025-08-18

**Authors:** Kiyoon Moon, Yosep Lee, Junseong Lee, Youngki Son, Youngje Woo, Eunju Jang, Sangseob Yun, Suncheol Park, Jangyong Kim

**Affiliations:** ^1^ Division of Vascular and Transplant Surgery, Department of Surgery, The Catholic University of Korea, Seoul, Republic of Korea; ^2^ Department of Cardiovascular Intervention Laboratory, Seoul St. Mary’s Hospital, The Catholic University of Korea, Seoul, Republic of Korea; ^3^ Department of Healthcare & Artificial Intelligence, The Catholic University of Korea, Seoul, Republic of Korea

**Keywords:** abdominal aortic aneurysm, computational fluid dynamics, hemodynamics, rupture, wall shear stress

## Abstract

**Background:**

Abdominal aortic aneurysm (AAA) rupture is a life-threatening event traditionally predicted by aneurysm diameter. However, many clinical observations have revealed that rupture can occur even in small aneurysms, suggesting the influence of additional biomechanical factors such as hemodynamics. The aim of this case series was to perform computational fluid dynamics (CFD) analyses based on CT scans of patients with confirmed abdominal aortic aneurysm rupture and to evaluate correlations between rupture sites and hemodynamic factors derived from simulations.

**Methods:**

This study analyzed four patients with confirmed ruptured fusiform infrarenal AAAs. Three-dimensional patient-specific models were reconstructed from CT scans and simulated using SimVascular, an open-source CFD platform. Simulations incorporated pulsatile inlet flow and three-element Windkessel outlet boundary conditions to calculate the following key hemodynamic parameters: time-averaged wall shear stress (TAWSS), oscillatory shear index (OSI), endothelial cell activation potential (ECAP), and relative residence time (RRT). Rupture sites were compared with spatial distributions of these parameters. Intraluminal thrombus (ILT) regions were estimated by overlaying flow lumen boundaries with the aneurysmal wall.

**Results:**

Rupture consistently occurred in regions of low TAWSS, high OSI, elevated ECAP, and high RRT. These sites also showed flow stagnation during systole and recirculation during diastole. ECAP demonstrated the highest spatial specificity for rupture. Overlay models revealed that ILT-prone zones corresponded with high-RRT regions and often co-localized with rupture sites.

**Conclusions:**

CFD-derived hemodynamic parameters, particularly ECAP was spatially correlated with AAA rupture sites. These findings support the utility of CFD in identifying rupture-prone regions and suggest its potential as a supplementary tool for risk stratification beyond diameter-based criteria.

## 1 Introduction

Abdominal aortic aneurysm (AAA), one of the major aortic diseases, affects approximately 0.9% of the global population (1). Its prevalence increases with age, particularly after the age of 60 (1–3). In South Korea, the incidence of AAA continues to rise due to population aging, with over 13,000 cases diagnosed in 2022, including about 5% rupture cases (4).

The risk of AAA rupture is primarily assessed based on aneurysm diameter, with repair generally recommended when the diameter exceeds 5.5 cm or grows rapidly (5, 6). However, in clinical practice, ruptures can occur in small aneurysms, and large aneurysms may remain stable, suggesting that factors beyond size contribute to rupture risk. Recent studies highlight the importance of hemodynamic characteristics in understanding aneurysm behavior (7–10).

Computational fluid dynamics (CFD) is a field that mathematically models physical phenomena of fluids and solves them numerically using computational processes. CFD has been widely applied in various industries, including aerospace, automotive engineering, and biomedical research, to analyze fluid behavior under different conditions. In the medical field, CFD plays a crucial role in understanding complex blood flow patterns, aiding in the diagnosis and treatment of cardiovascular diseases (11–13). One of the key advantages of CFD is its ability to provide detailed hemodynamic data in a non-invasive manner, making it a valuable tool for studying vascular diseases such as aortic aneurysms. Given that aneurysm rupture is not solely determined by size, there is a clear need to incorporate hemodynamic analysis into risk stratification (14–16). Computational fluid dynamics (CFD) offers a promising approach to evaluate these biomechanical factors.

However, despite its potential, CFD modeling has limitations. Validation of simulation results against actual physiological outcomes is essential but challenging. While previous studies have demonstrated concordance between CFD-predicted regions and markers of wall weakening or thrombus formation, few have investigated the spatial relationship between CFD-derived parameters and actual rupture sites confirmed by imaging. Therefore, the clinical translatability of CFD in rupture risk stratification remains an area of active investigation.

This study aimed to evaluate whether CFD-based hemodynamic analysis can localize rupture-prone regions in abdominal aortic aneurysms (AAA) by comparing simulation-derived parameters with CT-confirmed rupture sites. Rather than serving as a broad validation of CFD technology, this investigation focuses on a small clinical cohort to explore whether disturbed flow patterns correspond spatially to actual rupture points.

## 2 Materials and methods

### 2.1 Patient selection

Four patients who underwent aneurysm repair operation (surgical repair or endovascular repair) for ruptured AAA at Seoul St. Mary's Hospital from 2018 to 2023 were included in this study. All these patients had fusiform aneurysms only at the infra-renal abdominal aorta. The ruptured status of their aneurysms was confirmed by leakage of contrast with presence of hematoma surrounding the aneurysm on the CT scan. Patients without enhanced CT performed before urgent repair were excluded. Other aneurysm types (mycotic, traumatic) and post-EVAR cases were excluded to maintain a uniform pathology for comparison. All patients had 5 mm cut CT scans without pre-existing 3D reconstruction images. These scans were obtained as part of routine clinical evaluation prior to rupture confirmation. While thinner slices (<2.5 mm) are preferable for higher geometric fidelity, the available data provided sufficient resolution for generating accurate lumen boundaries and simulating general flow patterns. To compare simulation results with actual rupture sites, CT scans were reconstructed into three-dimensional models using 3D Slicer (Harvard Medical School, Boston, MA, USA), an open-source 3D reconstruction program. The rupture site was marked on axial CT images. Its corresponding location was identified on volume-rendered 3D images.

### 2.2 3D-modeling and meshing

Each AAA was reconstructed into a 3D model using CT images with the open-source CFD software program “SimVascular” (University of California, San Diego, CA, USA). Manual segmentation of vessels was performed for axial cut sections of CT images to define AAA contours from celiac axis level to common iliac artery bifurcation level. All aortic branches were excluded to reduce computational complexity and focus on the primary flow within the aneurysmal lumen. This approach may affect secondary flow dynamics, but prior studies have shown it provides reasonable accuracy in global wall shear stress analysis. Segmented contours were lofted to generate a 3D model, followed by necessary modifications and smoothing to finalize the model for analysis. The mesh for each patient-specific model was generated using the TetGen algorithm within SimVascular, with the global mesh edge size automatically determined by the software. The resulting meshes consisted of approximately 700,000–2,500,000 elements and 100,000–400,000 nodes, with minimum, maximum, and average element sizes of approximately 0.03 mm, 0.2 mm, and 0.12 mm, respectively.

### 2.3 Simulation

Three-dimensional incompressible Navier-Stokes equations in vascular models were solved with the finite volume method using the svSolver (University of California, San Diego, CA, USA) to analyze hemodynamic characteristics of AAAs. Blood was assumed to be an incompressible Newtonian fluid with a density of *ρ* = 1.06 g/cm^3^ and a dynamic viscosity of *μ* = 0.04 Pa·s. This assumption is justified based on the relatively high shear rates typically present in large-vessel arterial flows such as abdominal aortic aneurysms. Previous studies have supported the validity of this approximation in similar CFD analyses (7, 17). Patient-specific inlet flow measurements were not available due to the retrospective nature of this study. Therefore, a physiologically realistic pulsatile velocity waveform with a 1-second cardiac cycle was applied at the inlet using a prescribed velocity file derived from a typical resting cardiac output. The mean volumetric flow rate was approximately 5.4 L/min, closely reflecting baseline physiological conditions. An initial pressure of 100 mmHg was assigned as a baseline condition for numerical stability. This value does not correspond directly to either systolic or mean arterial pressure. No pulse pressure input was directly imposed, as the simulation used velocity-driven inlet conditions. Vessel walls were assumed to be rigid due to computational constraints and the difficulty of accurately estimating patient-specific wall elasticity. The time step size was set to 0.002 s. The outlet boundary conditions were modeled using a three-element Windkessel (RCR) configuration to simulate downstream vascular resistance and compliance. In the absence of patient-specific flow or pressure measurements, R, C, and Rd values were adopted from the publicly available Normal Aorto-femoral Model provided by the SimVascular project, which represents physiologically realistic values derived from a healthy aortoiliac anatomy. Although not patient-specific, these standardized parameters allowed for consistent hemodynamic comparison across cases. A total of 10 cardiac cycles were simulated. Data from the last two cycles were extracted and analyzed.

### 2.4 Post-processing

All simulation data were visualized using a post-processing program called ParaView (Kitware Inc., Clifton Park, NY, USA). First, Time-Averaged Wall Shear Stress (TAWSS) and Oscillatory Shear Index (OSI) were visualized from time-average data. For each individual AAA, quartile values of TAWSS were additionally calculated for the entire vessel wall and for the rupture site and subsequently compared. Endothelial Cell Activation Potential (ECAP) and Relative Residence Time (RRT) derived from TAWSS and OSI were also calculated and visualized. Mechanical parameters are provided in Equations (1, 2, 3), and (4):

(1) $$TAWSS=\;\frac{1}{T}\overset{T}{\underset{0}{\int }}\;|WSS|\;dt$$

(2) $$OSI=\frac{1}{2}\left(1-\frac{|\frac{1}{T}\int_{0}^{T}\;WSS\;dt|}{\frac{1}{T}\int_{0}^{T}\;|WSS|dt}\right)$$

(3) $$ECAP=\frac{OSI}{TAWSS}$$

(4) $$RRT=\;\frac{1}{(1-2\;\times \;OSI)\times \;TAWSS}$$

In addition, velocity fields were extracted and visualized at both peak systolic and end-diastolic phases of the cardiac cycle to evaluate phase-specific flow characteristics within the aneurysmal sac. This approach enabled identification of localized flow disturbances and recirculation zones, particularly in relation to anatomical locations of rupture sites.

To qualitatively validate the CFD results, we compared the regions of low TAWSS obtained from simulation with the actual rupture locations identified on CT imaging. This spatial correlation served as an indirect qualitative validation, supporting the physiological relevance of the simulated hemodynamic parameters in the context of aneurysm rupture.

To further investigate the spatial relationship between intraluminal thrombus (ILT) and RRT, we created an overlay model combining the full aneurysm wall geometry with the actual flow lumen reconstructed from the velocity field. This allowed for visual estimation of ILT regions, assumed to be the space between the aneurysm boundary and the perfused lumen. The resulting ILT overlay was then qualitatively compared with the spatial distribution of RRT.

**Figure 1:**
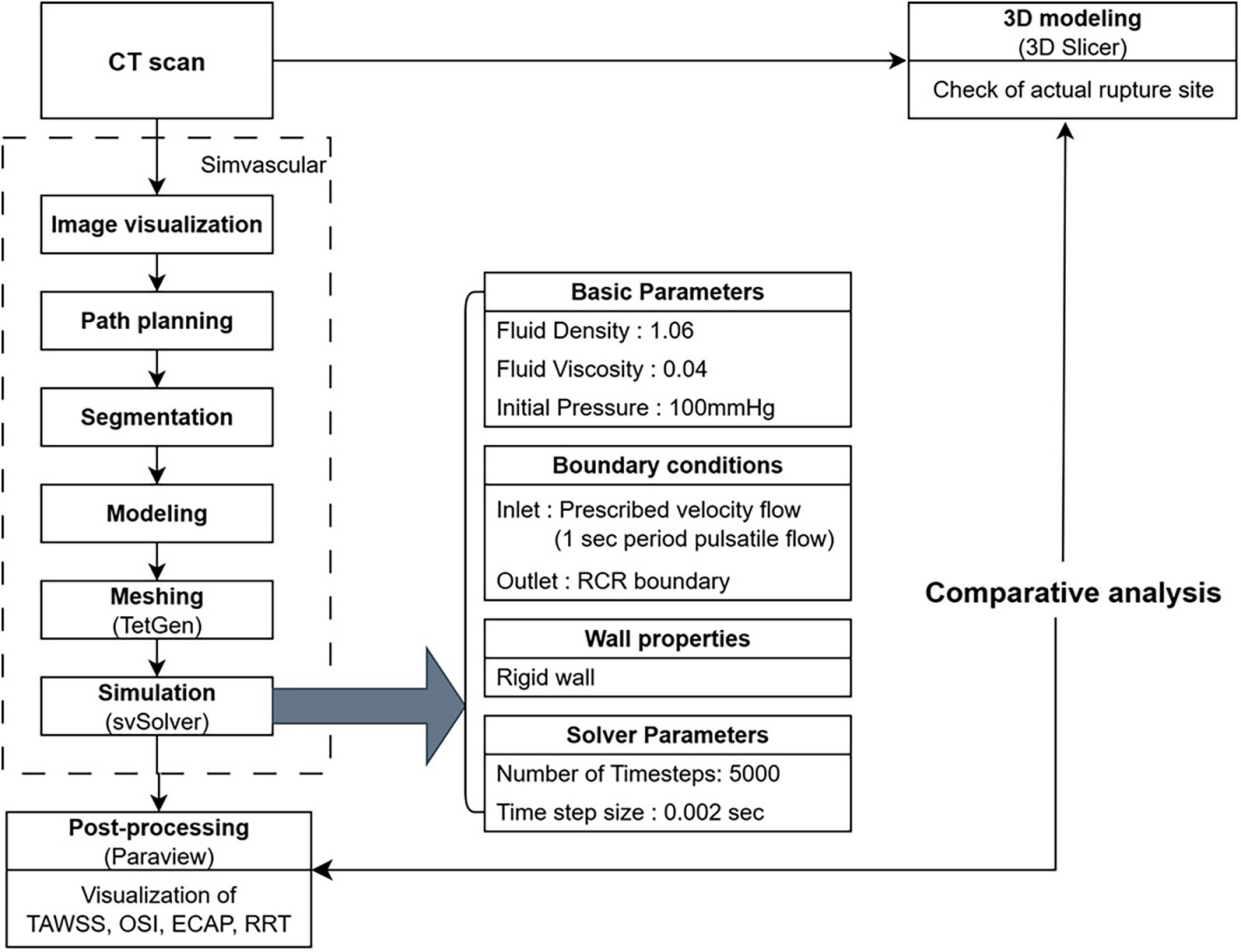
Workflow of computational fluid dynamic simulation and validation using CT imaging and post-processing.

A schematic summary of the overall workflow, including CT image acquisition, 3D reconstruction, mesh generation, simulation setup, and post-processing steps, is presented in Figure 1.

## 3 Results

This study included four patients (three males and one female) diagnosed with ruptured fusiform infrarenal AAA. Their mean patient age was 72.5 years and their mean maximum aneurysm diameter was 8.35 ± 2.38 cm. The maximum diameters of the four AAA cases were 6.2 cm, 9.8 cm, 11.4 cm, and 6 cm, respectively. All cases exceeded the typical repair threshold of 5.5 cm. Although maximum aneurysm diameter varied across cases, no clear correlation was observed between diameter and the spatial distribution of hemodynamic risk parameters. Interestingly, in cases 3 and 4, the rupture occurred at regions that were not the points of maximal diameter. This observation reinforces the notion that vessel geometry alone may not fully account for rupture risk.

**Figure 2:**
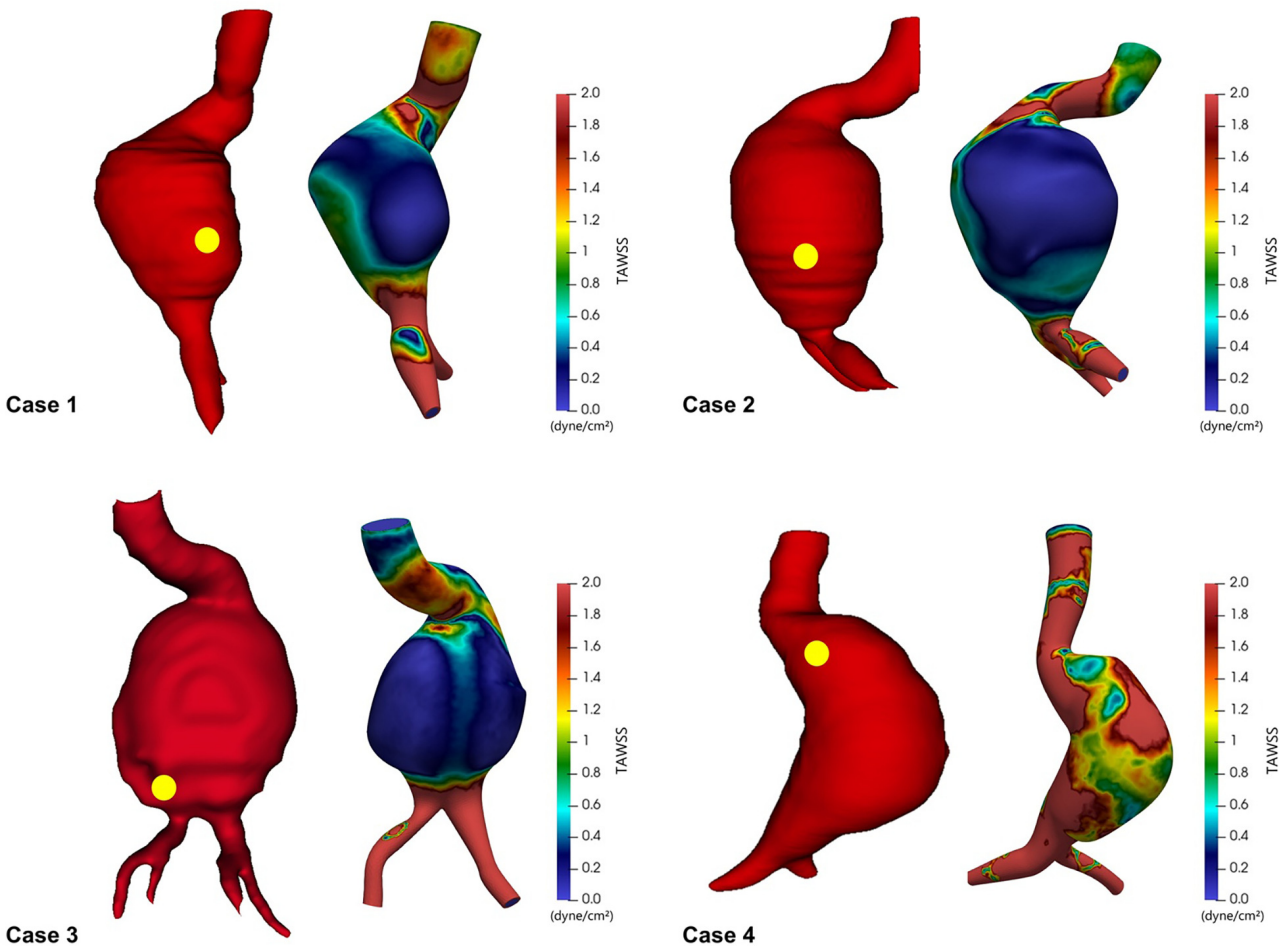
Time-averaged wall shear stress (TAWSS) distribution in ruptured abdominal aortic aneurysms, with yellow dots indicating actual rupture sites.

To evaluate relationships between hemodynamic forces and rupture locations, we first visualized TAWSS across the entire aortic wall and overlaid actual rupture sites as identified on preoperative CT images (Figure 2). The spatial correlation between regions of low TAWSS and rupture sites was evident in all four cases. In each patient, the rupture occurred in a region where the TAWSS was markedly reduced relative to the surrounding aneurysmal wall. These observations suggest that regions with reduced shear stress might represent biomechanically vulnerable zones that are more susceptible to aneurysmal wall rupture.

**Figure 3:**
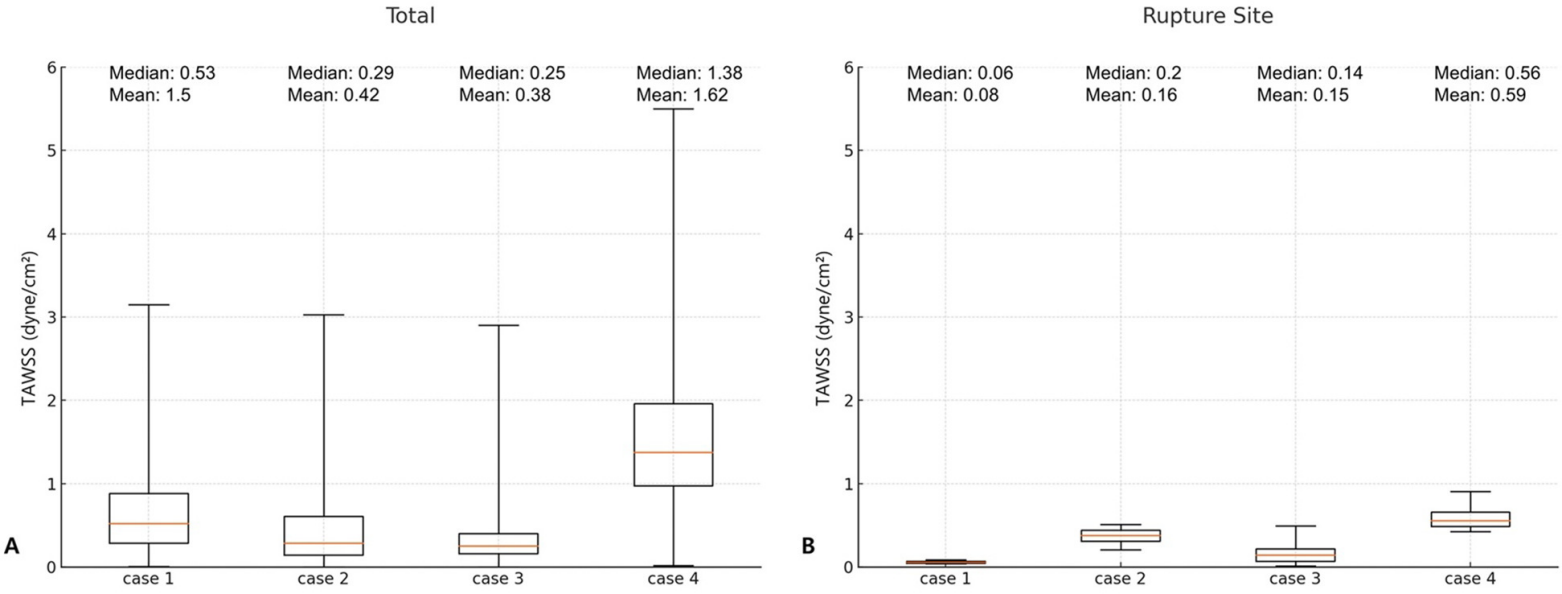
Comparison of time-averaged wall shear stress (TAWSS) in the entire aneurysm wall **(A)** and at the rupture site **(B)**.

To quantitatively support these visual findings, we conducted a box plot analysis comparing the distribution of TAWSS within the entire aneurysmal sac vs. those at rupture sites (Figure 3). This analysis consistently revealed the following pattern across all four cases: TAWSS values were lower at rupture sites than in the aneurysmal wall as a whole. While the mean TAWSS across the entire aneurysm ranged from 0.38 to 1.62 dyne/cm^2^, the corresponding mean TAWSS at the rupture locations ranged from 0.08 to 0.59 dyne/cm^2^. Similarly, median TAWSS values followed the same trend of reduction at rupture sites, underscoring the reproducibility of such association. These findings consistently demonstrated that rupture sites were located in regions of significantly lower TAWSS compared to the surrounding aneurysmal wall, supporting a potential link between reduced wall shear stress and rupture location.

**Figure 4:**
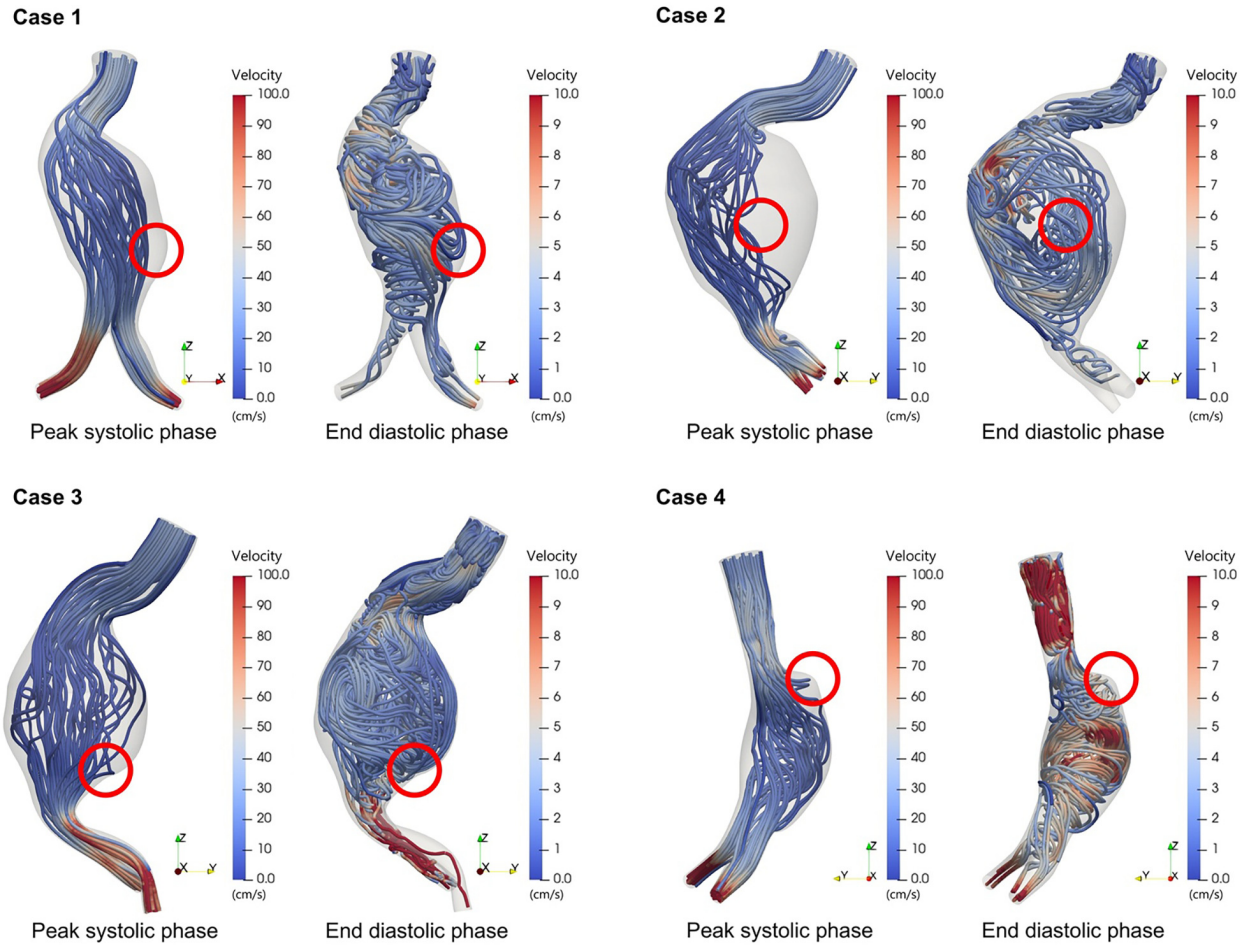
Flow patterns during peak systolic phase and end diastolic phase in ruptured abdominal aortic aneurysms.

To further investigate the flow dynamics at rupture locations, we visualized velocity streamlines during both peak systolic and end-diastolic phases of the cardiac cycle (Figure 4). In all four cases, the rupture site was located in a region that exhibited marked flow stagnation during systole—with minimal or absent forward flow—and prominent recirculatory flow during diastole. To address potential visualization artifacts due to streamline seeding, especially in regions of flow stagnation, seed points were adjusted to more accurately reflect velocity fields in complex aneurysmal geometries. These phase-dependent flow transitions were consistently observed at the rupture sites. Notably, these disturbed flow regions spatially corresponded with areas of low TAWSS, suggesting that chronic exposure to flow stagnation and vortex-induced shear oscillation may contribute to progressive wall weakening and rupture.

**Figure 5:**
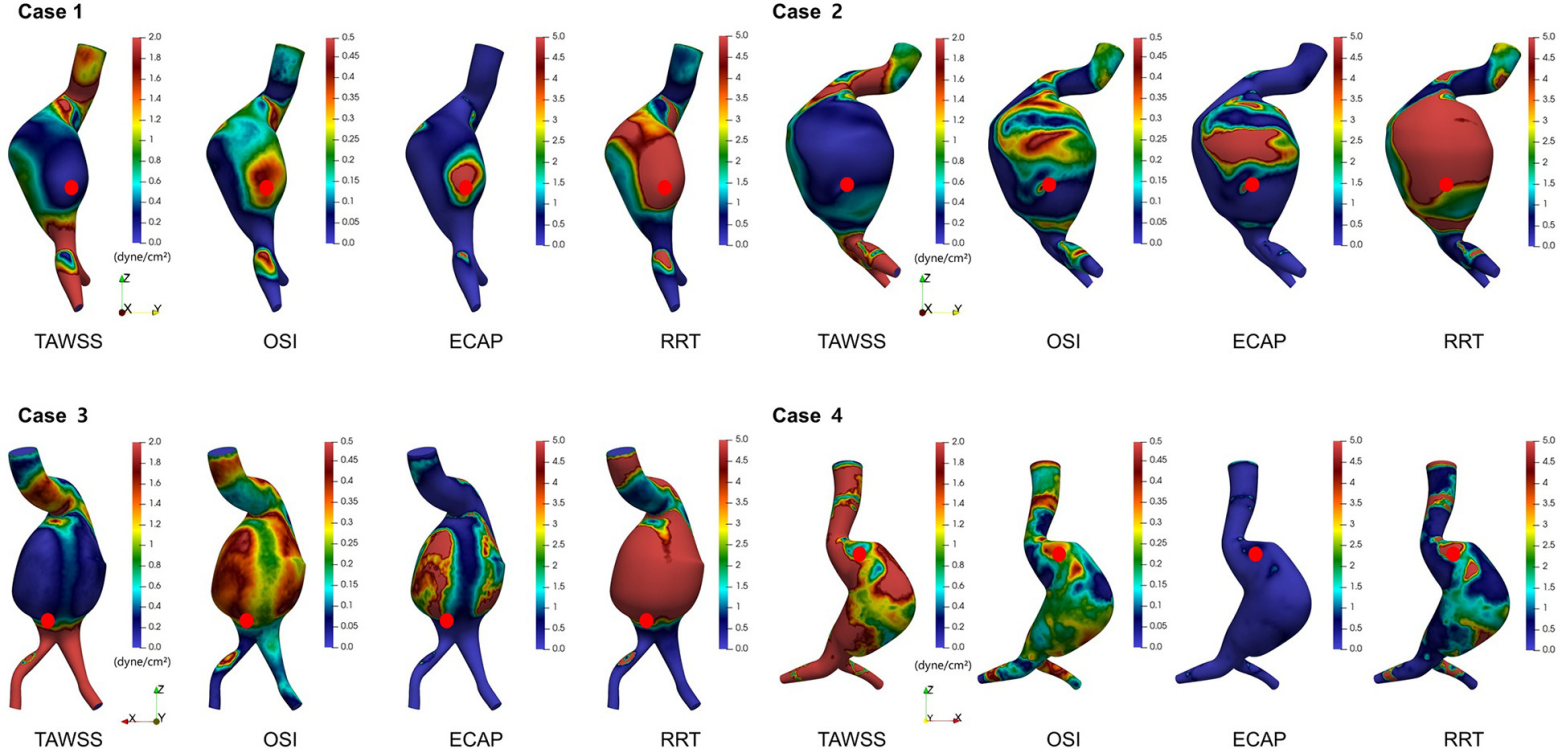
Spatial distributions of time-averaged wall shear stress (TAWSS), oscillatory shear index (OSI), endothelial cell activation potential (ECAP), and relative residence time (RRT) in ruptured abdominal aortic aneurysms.

In addition to TAWSS, we evaluated three other hemodynamic indices known to influence endothelial function and wall remodeling: OSI, ECAP, and RRT. For each patient, these indices were mapped across the aneurysm surface. Rupture sites were marked with red dots for clarity (Figure 5). In all four cases, rupture sites coincided with regions characterized by low TAWSS, elevated OSI, increased ECAP, and high RRT. Although additional regions with similarly elevated values were occasionally observed they did not correspond to rupture sites. This suggests that while these hemodynamic parameters are indicative of vulnerability, they may not be exclusively predictive of rupture. Among the parameters, ECAP exhibited the strongest spatial correspondence with rupture locations, suggesting its potential as a particularly informative marker of endothelial dysfunction and biomechanical stress. While ECAP has been associated with thrombus formation in previous studies, a strong spatial overlap with rupture sites was observed in this study. Taken together, these CFD-derived findings suggest that regions characterized by low shear stress, high oscillatory flow, and prolonged particle residence may experience altered mechanical environments that contribute to localized wall weakening and increased susceptibility to rupture.

**Figure 6:**
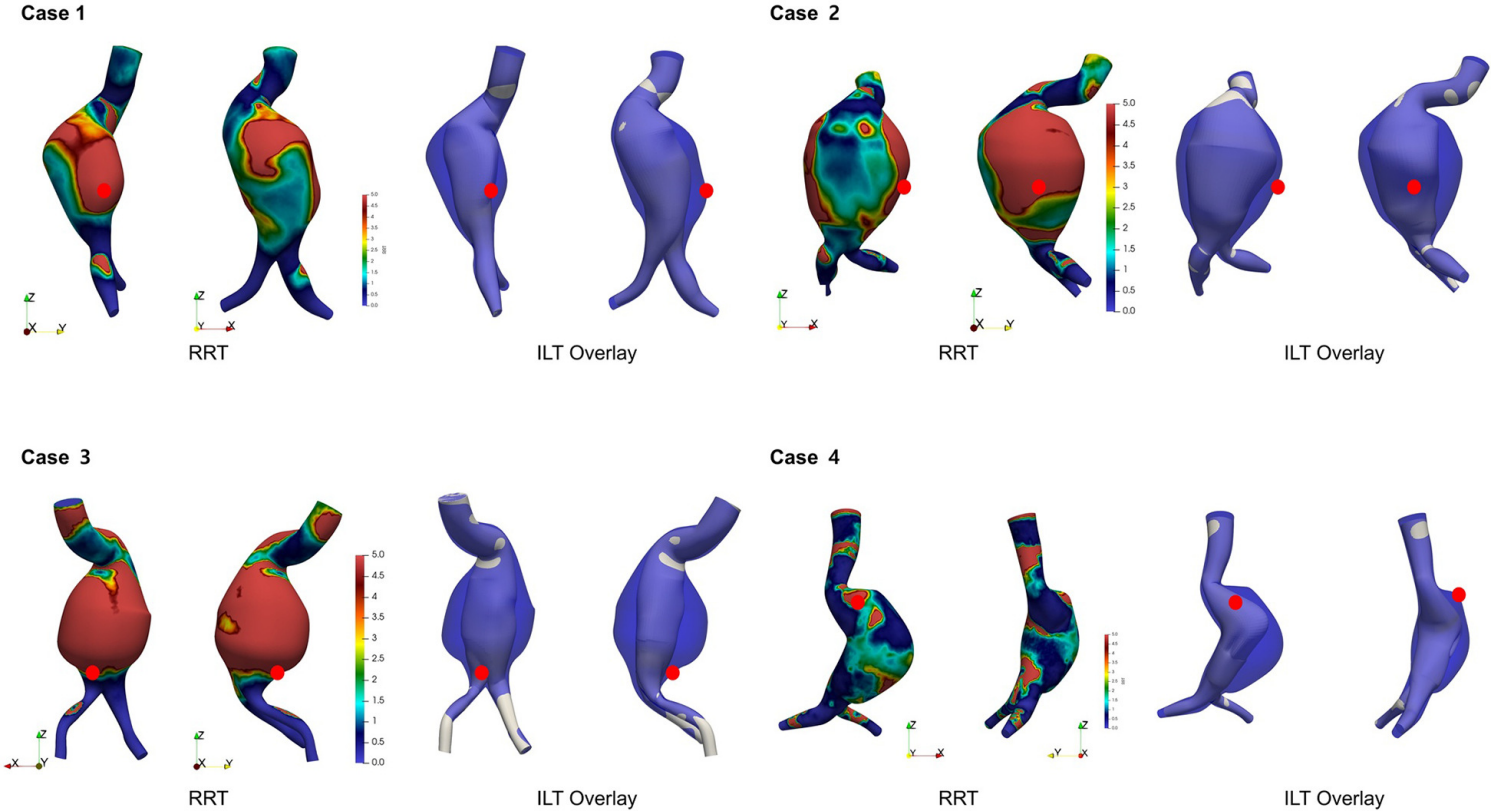
Visualization of presumed intraluminal thrombus (ILT) regions based on aneurysm wall and flow lumen overlay. High RRT regions overlap with ILT and rupture sites (red dot).

Figure 6 demonstrates the spatial overlay between the aneurysm wall and the flow-perfused lumen, highlighting regions of presumed ILT. These ILT-prone areas, located between the outer aneurysm contour and the active flow channel, showed strong spatial correspondence with regions of elevated RRT. RRT was not uniformly distributed; instead, it tended to be elevated in areas with flow stagnation or recirculation, particularly along the posterior or lateral walls of the aneurysmal sac. These regions overlapped with presumed ILT zones. In all four cases, rupture sites were located within or adjacent to these high-RRT and ILT regions.

## 4 Discussion

AAA rupture is a biomechanical event that occurs when the expanding vessel wall exceeds its tensile strength. According to Laplace's law, in a hollow cylindrical structure such as the aorta, wall tension is directly proportional to both the internal pressure and the vessel radius (7). Consequently, as the aneurysmal diameter increases, wall stress also increases proportionally, thereby elevating the risk of rupture (14). However, clinical and experimental observations have shown that AAA rupture does not necessarily occur at the site with the maximal diameter. This indicates that rupture is not merely a consequence of elevated pressure or size alone. Rather, rupture occurs when blood flow-induced wall stress exceeds the limit of aortic wall tissue strength (15, 16). The abdominal aorta does not have a uniform shape in cases with aneurysmal changes. The dilation is often irregular rather than having a smooth, elliptical expansion. As a result, both the wall stress and the strength of the aortic tissue are not uniform throughout the aorta. Therefore, regional variations in aortic wall properties should be considered when evaluating rupture risk in AAA.

Various hemodynamic factors have been identified in relation to vascular wall properties. Among these, wall shear stress (WSS) is considered one of the most critical as it represents the tangential frictional force exerted on the vessel wall by blood flow. Numerous studies have demonstrated an association between WSS and AAA rupture (9, 18–20). Under physiological conditions, WSS is typically maintained at approximately 15–20 dyne/cm^2^. Endothelial cells in the aortic wall maintain an anti-inflammatory and anti-thrombotic state when they sense this level of WSS. However, when WSS decreases, endothelial cells will fail to receive adequate mechanical stimulation, leading to loss of normal function. Inflammatory responses in the aortic wall are known to be regulated by the ratio of macrophage type I (classically activated, M1) to type II (alternatively activated, M2) (21). M1 macrophages can promote pro-inflammatory responses and matrix degradation, whereas M2 macrophages possess anti-inflammatory effects and pro-fibrotic activity. A decrease in WSS has been shown to enhance M1 macrophage differentiation via interferon-gamma, subsequently leading to the secretion of pro-inflammatory cytokines such as TNF-α, IL-1β, and IL-6. These cytokines can initiate a strong inflammatory cascade and induce the release of matrix metalloproteinases, contributing to extracellular matrix degradation (21, 22). Findings of this study are consistent with these mechanisms. Overall, WSS within the aneurysm sac was lower than that in non-aneurysmal regions, with the rupture site exhibiting particularly reduced WSS compared to other regions.

Regions with low WSS were found to correspond to areas with little to no forward flow during the peak systolic phase and recirculating flow patterns during the end-diastolic phase. This flow pattern was consistently observed at the rupture sites across all cases (Figure 4). These recirculation zones are characterized by a flow that either moves in the reverse direction or forms slow-moving vortices, resulting in stagnation. Consequently, the velocity of blood in contact with the vessel wall is markedly reduced in these areas, leading to a significant decrease in WSS (10, 23).

In addition to WSS, several other hemodynamic factors have been identified as being associated with AAA rupture. Among them, the OSI, ECAP, and RRT are well-recognized parameters. These parameters often demonstrate overlapping spatial patterns, which aligns with the mathematical relationships inherent in the calculation of ECAP and RRT. OSI quantifies temporal oscillation of WSS direction relative to predominant axial flow. Elevated OSI values indicate frequent changes in flow direction. They are known to induce pathological activation of endothelial cells. While OSI reflects the degree of oscillation in shear direction, it is inherently a bidirectional metric and does not fully capture the multidirectional complexity of wall shear stress vectors across the aneurysmal surface. Therefore, OSI may underestimate areas with complex rotational or turbulent shear behavior. Nevertheless, Chen et al. demonstrated that regions with elevated OSI and RRT in spontaneously hypertensive rats were closely associated with marked arterial remodeling and increased stiffness. Importantly, these aberrant hemodynamic patterns persisted even after normalization of systemic blood pressure, implying that sustained flow disturbances may have originated from structural alterations in the vessel wall rather than pressure elevation alone (24). ECAP defined as the ratio of OSI to TAWSS reflects areas of disturbed flow where a high oscillatory shear coincides with a low shear magnitude. Consequently, higher ECAP values were observed in regions with elevated OSI and reduced TAWSS. This parameter has been suggested as a more sensitive indicator of pathological endothelial activation (18, 23). Including ECAP and RRT allows for a more comprehensive characterization of disturbed hemodynamics, improving the ability to localize rupture-prone regions beyond what is possible with TAWSS and OSI alone. Similarly, the RRT, which quantifies the duration that blood particles remain near the vessel wall, is also derived from OSI and TAWSS. RRT values are elevated in recirculation areas where blood flow is slow as previously described. RRT, like other hemodynamic parameters, is associated with endothelial dysfunction. However, previous studies have reported that RRT is more directly linked to an increased risk of thrombus formation rather than to endothelial dysfunction itself. In this study, areas with elevated RRT values were found to correspond predominantly to regions with ILT deposition. The mechanism of AAA rupture is thought to involve not only weakening of the aortic wall due to shear stress reduction from altered hemodynamics, but also direct impact of ILT itself, which has been shown to contribute to rupture risk.

The impact of ILT on AAA rupture remains controversial. While some studies have reported that ILT contributes to aortic wall weakening and thereby increases the risk of rupture, others have suggested a potentially protective effect. Specifically, certain investigations have proposed that ILT can exert a cushioning effect, attenuating the transmission of hemodynamic pressure to the aneurysmal wall and modifying local flow patterns in a manner that can reduce wall stress and rupture risk (25, 26). Conversely, the hypothesis that ILT promotes wall degeneration is primarily attributed to the “hypoxic damage” mechanism. It has been postulated that ILT can impede oxygen diffusion from the lumen to the aortic wall, resulting in a hypoxic microenvironment that contributes to progressive wall weakening (27, 28). Several studies have further demonstrated an inverse relationship between ILT thickness and wall strength, supporting the notion that larger thrombus burdens are associated with increased structural vulnerability (7, 28). ILT has also been shown to contain platelets, red and white blood cells, inflammatory cytokines, and proteolytic enzymes, which may diffuse into the underlying wall tissue and induce chronic inflammation (23, 25, 29, 30). This chronic inflammatory state may exacerbate extracellular matrix degradation and predispose the wall to rupture (26). Boyd et al. have reviewed morphologic characteristics of ILT and their hemodynamic implications and suggested that although ILT may initially confer a protective effect, it may become deleterious over time as it contributes to adverse remodeling and wall weakening (29). Longitudinal studies increasingly support the consensus that ILT contributes to an elevated risk of AAA rupture. Consistent with these findings, results of this study demonstrated that ruptures occurred in regions where ILT was present. As previously noted, thrombus formation was predominantly observed in regions exhibiting high RRT. Considering that rupture was observed within a subset of these high-RRT regions, our results suggest a potential association between ILT presence and increased AAA rupture risk.

This study has several limitations. First, it has a small sample size. Although a robust statistical analysis was not feasible, the observed consistency between CFD simulation results and actual rupture sites suggested a meaningful trend and supported the reliability of the model at a qualitative level. Second, the inlet and outlet boundary conditions were defined using generalized flow profiles rather than patient-specific measurements, and the vessel wall was assumed to be rigid. These simplifications were necessary due to the retrospective nature of the study and limitations in data availability. However, they may reduce the physiological accuracy of the simulation. Future studies should incorporate patient-specific flow data acquired from advanced imaging modalities such as 4D Flow MRI, phase-contrast MRI or duplex ultrasound. In addition, adopting fluid-structure interaction models that account for vessel wall compliance and mechanical behavior would improve the biomechanical fidelity of the analysis. Third, due to time constraints, a formal mesh convergence study was not performed. This may introduce numerical uncertainty, particularly in localized flow features. As this is a pilot study, future research will include mesh sensitivity analysis to improve numerical accuracy. Fourth, other hemodynamic metrics, such as transverse wall shear stress or turbulence intensity, were not assessed in this study but may offer additional insights and will be explored in future analyses. Lastly, while simulations could provide insight into hemodynamic features associated with rupture-prone regions, they could not offer quantitative thresholds or absolute risk values for predicting rupture with clinical certainty. This limitation reflects an inherent constraint of simulation-based studies and underscores the need for integration with clinical and biomechanical validation data. Despite these limitations, the present study provides meaningful evidence that rupture risk in abdominal aortic aneurysms might be influenced not only by aneurysm diameter, but also by underlying hemodynamic factors. This approach offers a noninvasive tool to enhance rupture risk stratification in AAA patients. With advances in imaging and modeling technologies, CFD simulation based on routine CT angiography could be integrated into clinical workflows. Such integration would allow for patient-specific risk assessment, particularly in cases with borderline aneurysm size or rapid morphological change. While further validation with larger datasets and prospective studies is required, this study provides foundational evidence that hemodynamic modeling could support more personalized surveillance and decision-making in AAA treatment.

## 5 Conclusions

This study demonstrates that AAA rupture is strongly associated with localized hemodynamic disturbances. Computational simulations revealed that rupture sites consistently corresponded to regions of low TAWSS, high OSI, elevated ECAP, and increased RRT—often accompanied by recirculating flow and ILT deposition. These findings suggest that CFD-based hemodynamic analysis can provide meaningful insight into rupture-prone areas and may enhance risk stratification in patients with AAA, particularly in cases where conventional size-based criteria alone are insufficient.

## Data Availability

The raw data supporting the conclusions of this article will be made available by the authors, without undue reservation.
