# Correction: Hemodynamic predictors of rupture in abdominal aortic aneurysms: a case series using computational fluid dynamics

**DOI:** 10.3389/fcvm.2025.1690884

**Published:** 2025-09-17

**Authors:** Kiyoon Moon, Yosep Lee, Junseong Lee, Youngki Son, Youngje Woo, Eunju Jang, Sangseob Yun, Suncheol Park, Jangyong Kim

**Affiliations:** ^1^ Division of Vascular and Transplant Surgery, Department of Surgery, The Catholic University of Korea, Seoul, Republic of Korea; ^2^ Department of Cardiovascular Intervention Laboratory, Seoul St. Mary’s Hospital, The Catholic University of Korea, Seoul, Republic of Korea; ^3^ Department of Healthcare & Artificial Intelligence, The Catholic University of Korea, Seoul, Republic of Korea

**Keywords:** abdominal aortic aneurysm, computational fluid dynamics, hemodynamics, rupture, wall shear stress

The figures were in the wrong order in the published article. Figures 4 and 6 were mistakenly interchanged. The order has now been corrected.

**Figure 4:**
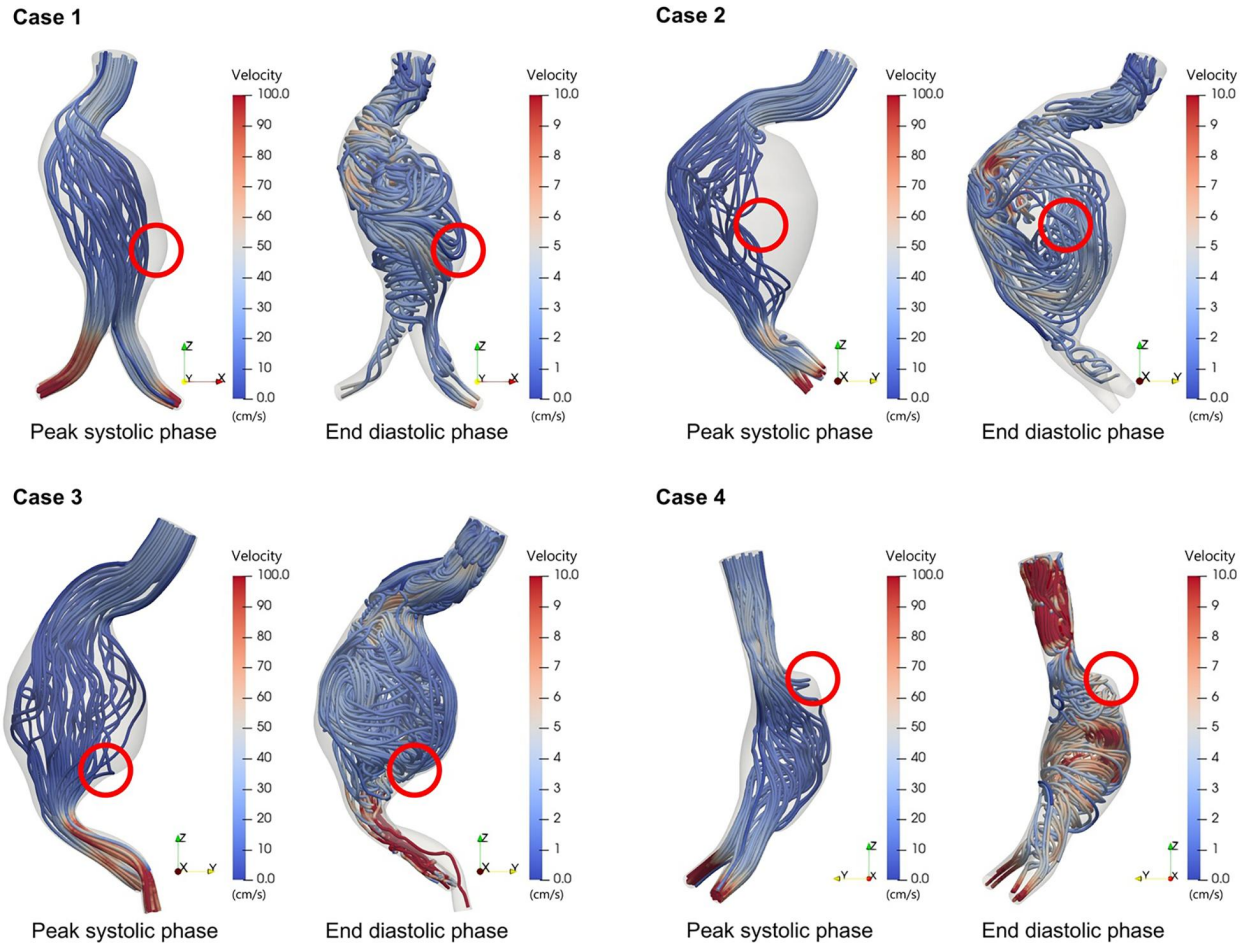
Flow patterns during peak systolic phase and end diastolic phase in ruptured abdominal aortic aneurysms.

**Figure 6:**
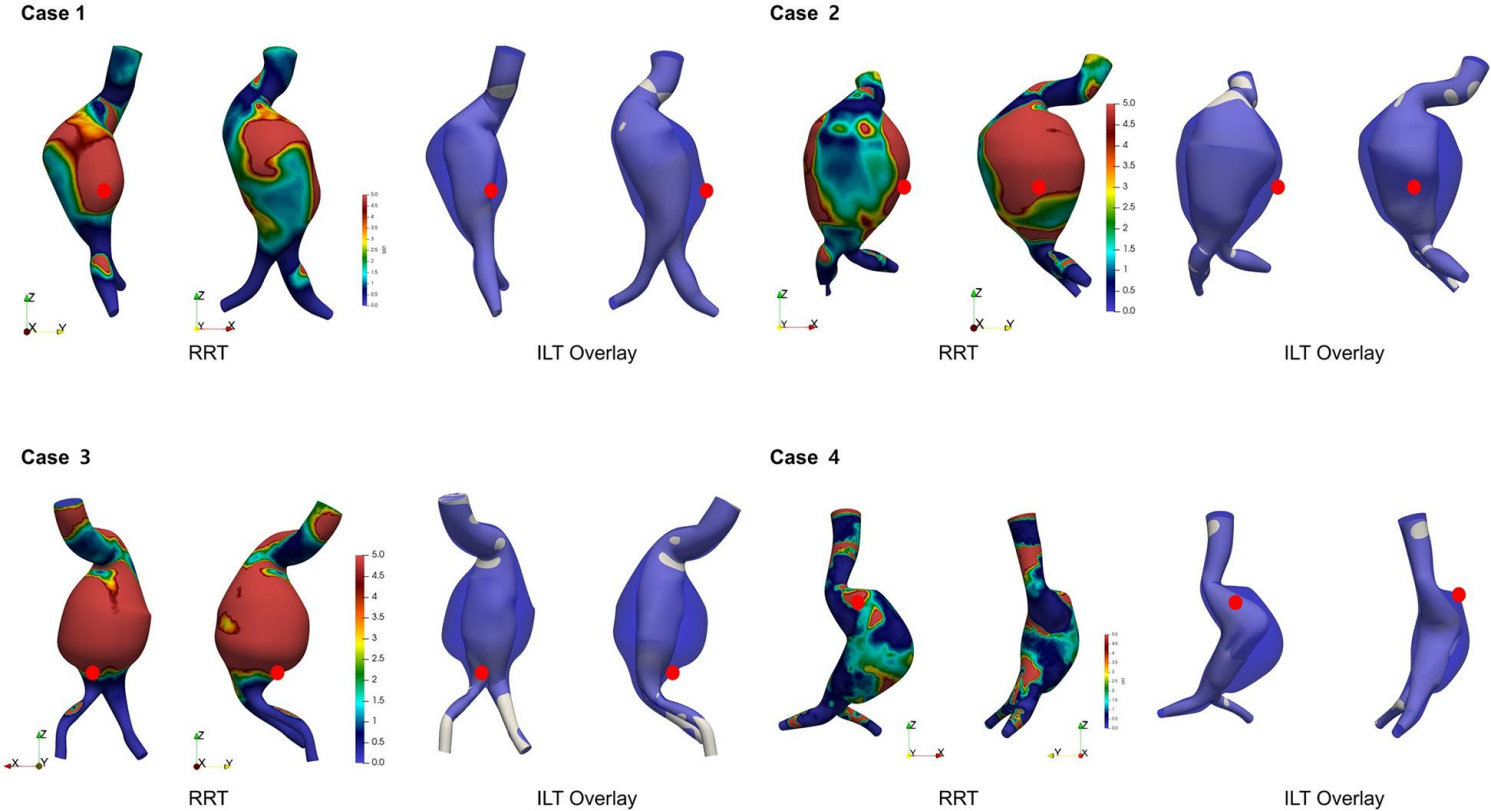
Visualization of presumed intraluminal thrombus (ILT) regions based on aneurysm wall and flow lumen overlay. High RRT regions overlap with ILT and rupture sites (red dot).

The original version of this article has been updated.

